# A new variant produced by *Rhizoctonia solani* AG1-IC isolate CH-1 with a new type of nuclei

**DOI:** 10.1186/s40529-014-0069-z

**Published:** 2014-09-25

**Authors:** Yun-Nung Tsai, Wen-Hsiung Ko

**Affiliations:** Department of Plant Pathology, National Chung Hsing University, Taichung, Taiwan

**Keywords:** Heterokaryon, Homokaryon, Protoplast, Rhizoctonia solani, Tuft formation, Variant

## Abstract

**Background:**

Isolate CH-1 of *Rhizoctonia solani* Kühn was commonly used in our studies of the pathogenicity and genetics of this pathogen. During the preparation of homokaryons through protoplast regeneration and tuft formation, a defective homokaryon was detected and a new variant was obtained.

**Results:**

When tuft formation was used to identify the karyotic nature of single protoplast regenerants (SPRs) of *Rhizoctonia solani* AG1-IC isolate CH-1, one homokaryon type designated as A type and the parental heterokaryon designated as AB type were obtained. The homokaryon B type was not found. Various approaches were used to obtain SPRs, including from fast or slow growing protoplast regenerants, and from regenerants of protoplasts released from mycelia grown in different nutrient broths or at different temperatures. Without exception, all these SPRs were either homokaryon A or heterokaryon AB. Moreover, the SPRs obtained from different generations of SPRs, and from different generations of SPRs treated with lytic enzymes 3 to 4 times also were invariably either homokaryon A or heterokaryon AB. When single hyphal isolates were obtained from the tuft resulting from the pairing between homokaryon A and heterokaryon AB, only the heterokaryon and a variant were obtained. The variant did not form tuft when paired with parental heterokaryon AB or homokaryon A. Its protoplast regenerants gave rise to heterokaryon AB, homokaryon A and the variant, indicating that it is a new kind of heterokaryon.

**Conclusion:**

Inability to obtain homokaryon B despite numerous attempts suggests that the B type nuclei are probably defective and are dependent on A type nuclei for their multiplication. This is the first report of a heterokaryotic *R. solani* strain carrying a defective type of nuclei. A new variant which is a new kind of heterokaryon was obtained from the tuft resulting from the paring between the homokaryon A and the parental heterokaryon AB.

**Electronic supplementary material:**

The online version of this article (doi:10.1186/s40529-014-0069-z) contains supplementary material, which is available to authorized users.

## Background

Protoplast released from mycelia of fungi have been used as experimental tools in physiological and biochemical studies and protoplast fusion as a means of establishing genetic crosses for genetic study and strain improvements (Peberdy [1989]). Recently, protoplasts of fungi and oomycetes also have been used in transfer of nuclei (Sivan et al. [1990]; Vagvolgyi and Ferenczy [1992]; Gu and Ko [1998], [2000a], [2001]; Wang et al. [2005]) and mitochondria (Gunge and Sakaguchi [1979]; Yoshida [1979]; Sulo et al. [1989]; Gu and Ko [2000b], [2005]; Ko [2007]). Organelle transfer of fungi and oomycetes is still in the early stage of development, and its possible application in the biological and genetical studies remains to be exploited.

*Rhizoctonia solani* Kühn is a widespread soil-borne plant pathogen causing economically important diseases in a wide range of crops (Anderson [1982]; Adams [1988]). It is desirable to apply organelle transfer to the studies of pathogenicity and genetics of this important fungal plant pathogen. A project was, therefore, initiated for the study of organelle transfer of the isolate CH-1 of *Rhizoctonia solani* AG1-IC commonly used in our research (Liu et al. [2010], [2011]; Tsai et al. [2012]). For organelle transfer, it is preferable to use homokaryotic isolates. In *R. solani*, homokaryotic isolates are normally derived from single basidiospores (Whitney and Parmeter [1963]) or protoplasts (Phillips [1993]). Since *R. solani* AG1-IC is heterothallic (Qu et al. [2008]) and our numerous attempts to induce basidiospore formation of isolate CH-1 also were not successful, protoplasts were used for preparation of homokaryons. In the process of homokaryon preparation, it was found that colonies derived from protoplasts of isolate CH-1 consisted of heterokaryon and one type of homokaryon only. The other type of homokaryon was assumed to be defective and not viable. Moreover, a new colony type variant was obtained from the tuft resulting from the pairings between the homokaryon and the heterokaryotic parent. Details of the study are reported herein.

## Methods

### Source of R. solani

Isolate CH-1 of *Rhizoctonia solani* AG1-IC was provided by Dr. J. W. Huang of the Department of Plant Pathology, National Chung Hsing University, Taichung, Taiwan. The Culture was deposited at the Bioresouce Collection and Research Center, Food Industry Research and Development Institute, Hsinchu, Taiwan (BCRC 34905), and maintained on potato dextrose agar (PDA).

### Protoplast formation

The method of Liu et al. ([2010]) was modified for protoplast formation and regeneration. A 5-mm culture disk of the fungus used was placed near the edge of a PDA plate (9 cm diam). After incubation at 24°C for 3 days, four culture disks were obtained from the advancing margin of the colony and placed on a cellophane disk (9 cm diam) laid on V-8 beef extract agar containing 1% V-8 juice, 0.1% beef extract and 2% agar. After incubation at 24°C for 12 h, the culture disks were cut off with a sterile scalpel and the cellophane disk was placed in 300 ml sterile distilled water in a 500-ml beaker. The mycelial mat was transferred to 25 ml V-8 broth consisting of 5% V-8 juice centrifuged at 1000 g for 5 min and adjusted to pH 6 with 1 N NaOH in a 250-ml-flask. After incubation at 24°C under light for 24 h, the mycelial mat with numerous young hyphae was transferred to a sterile 8-cm screen (Buckbee Mears, St. Paul, MN) with 150-μm pore size, rinsed with 200 ml sterile distilled water and placed in 5 ml stabilizer solution I (0.6 M manitol, 0.04 M CaCl_2_, and 0.01 M Tis-HCl, pH 7.5) mixed with 50 mg Drislase (D-9515, Sigma, St. Louis, MO, USA) and 25 mg lysing enzymes (L-1412, Sigma) in a 15-ml centrifuge tube. Stabilizer solution with enzymes was sterilized by filtration through 0.22 μm membrane filter (Millex, Millipore, Co Cork, Ireland). The centrifuge tube containing the mixture was incubated at 37°C for 15 min and 34°C for 105 min with gentle shaking to release protoplasts. Protoplasts were separated from non-digested mycelial fragments by filtration through a Whatman No. 113 filter paper (30-μm pore size) and the filtrate was collected in another centrifuge tube. To remove the lytic enzymes, 1 ml 0.8 M mannitol solution was pipetted onto the bottom of the centrifuge tube containing the protoplast suspension before centrifugation at 1000 g for 10 min. The supernatant containing the lytic enzymes was discarded and protoplasts retained in the bottom portion were used for regeneration.

### Preparation of single-protoplast regenerants (SPRs)

Protoplasts in the centrifuge tube were mixed with 1 ml of stabilizing solution II consisting of 0.1 M sucrose, 0.1 M CaCl_2_ and 0.1 M Ca(NO_3_)_2_, supplemented with 1% clarified V-8 juice. The mixture was equally dispensed into two 1.5-ml centrifuge tubes. After incubation at 24°C for 24 h in darkness, the concentration of regenerated protoplasts was adjusted to 2 to 3 protoplasts per 10-μl drop with a Pipetman microliter pipette (West Coast Scientific, CA, USA) (Ann et al. [2010]; Ko et al. [1973]). Twenty drops of the protoplast suspension were evenly distributed on a PDA plate. After incubation at 24°C for 24 h under light, colonies originated from SPRs were each transferred to a PDA slant under a dissecting microscope and stored at 24°C for subsequent study.

### Determination of karyotic nature

Karyotic nature of SPRs or single hyphal isolates was identified by tuft formation. At the initial test, 10 SPRs were each paired with the parental isolate on PDCYA consisting of PDA amended with 0.1% charcoal and 0.5% yeast extract modified from the medium described by Butler and Bolkan ([1973]), and incubated at 24°C under light for 4 days. Six SPRs did not form tuft indicating that they were heterokaryons just like the parental isolate, while 4 SPRs formed tuft indicating that they were homokaryons. When one of the homokaryons was paired separately with the other three homokaryons, none of the parings showed tuft formation, indicating that all of them belonged to the same type of homokaryon. Since *R. solani* AG1-IC has been reported to produce two types of homokaryons (Ou et al. [2008]), this type of homokaryon was designated as A, while the other not yet found homokaryon type was designated as B. The heterokaryon was designated as AB. Subsequently in search for the B type homokaryon, the karyotic nature of SPRs was determined by paring each SPR with an A type homokaryon and the parental heterokaryon AB. The B type homokaryon should form tuft when paired with A type homokaryon and heterokaryon AB.

### Isolation of single hyphal isolates from tuft

Homokaryon A and heterokaryotic parent were paired on PDCYA as described for tuft formation. After incubation at 24°C for 3 days, mycelial mat was obtained from the tuft with a sterile scalpel and placed on the center of a plate containing 2% water agar. After incubation at 24°C for 24 h, single hyphal tip isolates were isolated as described by Whitney and Parmeter ([1963]), and were each transferred to a PDA plate for colony formation.

## Results

### Confirmation of karyotic nature of SPRs

During the initial test, the 10 SPRs obtained consisted of 4 putative homokaryon A and 6 putative heterokaryon AB based on the tuft formation in the pairing with parental isolate. Randomly selected 3 putative homokaryon A and 3 putative heterokaryon AB were subjected to protoplast formation and regeneration, and the karyotic nature of each SPR was determined by tuft formation in pairing with the parental isolate. All 3 SPRs of putative homokaryon A produced only homokaryon A type SPRs (Table 1), confirming that they were true A type homokaryon. All the 3 SPRs of putative heterokaryon AB produced both homokaryon A type and heterokaryon AB type SPRs, confirming that they were true heterokaryon AB.

**Table 1:** **Karyotic nature of regenerants of protoplasts of** ***Rhizoctonia solani*** **AG1-IC isolate CH-1 from putative homokaryons and putative heterokaryons**

Protoplast origin	Karyotic nature*(no.)			Total
	A	B	AB	
Putative homokaryon A				
1	25	0	0	25
2	25	0	0	25
3	25	0	0	25
Putative heterokaryon AB				
1	15	0	10	25
2	8	0	17	25
3	9	0	16	25

*A, homokaryon A; B, homokaryon B; AB, heterokaryon AB.

### Searching for B type homokaryon

During the initial search, five independent experiments were carried out and ranging from 16 to 48 SPRs per experiment were obtained. All the experiments produced A type homokaryon and heterokaryon AB with the exception of Exp. 4 which produced only heterokaryon AB (Table 2). Since all five experiments failed to produce B type homokaryon, one more experiment was carried out to generate a total of 80 SPRs. Again only A type homokaryon and heterokaryon AB were obtained.

**Table 2:** **Karyotic nature of protoplast regenerants of** ***Rhizoctonia solani*** **AG1-IC isolate CH-1**

Experiment	Karyotic nature*(no.)			Total
	A	B	AB	
1	6	0	20	26
2	8	0	40	48
3	10	0	15	25
4	0	0	16	16
5	14	0	10	24
6	34	0	46	80
Total	72	0	93	165

*A, homokaryon A; B, homokaryon B; AB, heterokaryon AB.

To test whether fast or slow growth was the reason for failure to obtain B type homokaryon, SPRs were isolated after 10 or 72 h regeneration in comparison with the original 24 h regeneration. All the tests gave only A type homokaryon and heterokaryon AB (Table 3). To test if certain nutrient may favor the multiplication of B type nuclei, the fungus was grown in 10% V-8 juice broth or potato dextrose broth supplemented with 0.1% yeast extract in comparison with the original 5% V-8 juice broth before enzyme treatment to release protoplasts. All SPRs obtained were still either A type homokaryon or heterokaryon AB (Table 4). To test if high or low temperature may encourage the multiplication of B type nuclei, the fungus was grown at 12 or 32°C in comparison with the original 24°C before enzyme treatment for release of protoplasts. All SPRs obtained in each treatment were similar, containing only A type homokaryon and heterokaryon AB (Table 5).

**Table 3:** **Karyotic nature of protoplast regenerants of** ***Rhizoctonia solani*** **AG1-IC isolate CH-1 obtained from different regeneration times**

Regeneration time (h)	Karyotic nature*(no.)			Total
	A	B	AB	
10	4	0	6	10
24 (original)	7	0	13	20
72	8	0	17	25

*A, homokaryon A; B, homokaryon B; AB, heterokaryon AB.

**Table 4:** **Karyotic nature of regenerants of protoplasts of** ***Rhizoctonia solani*** **AG1-IC isolate CH-1 obtained from mycelia grown in different nutrient broths**

Nutrient broth	Karyotic nature*(no.)			Total
	A	B	AB	
PDB + 0.1% yeast extract	5	0	26	31
10% V-8 juce	4	0	28	32
5% V-8 juice (original)	5	0	33	38

*A, homokaryon A; B, homokaryon B; AB, heterokaryon AB.

**Table 5:** **Karyotic nature of regenerants of protoplasts of** ***Rhizoctonia solani*** **AG1-IC isolate CH-1 obtained from mycelial mat incubated at low or high temperature**

Temperature	Karyotic nature*(no.)			Total
	A	B	AB	
12	16	0	3	19
24 (original)	9	0	10	19
32	5	0	15	20

*A, homokaryon A; B, homokaryon B; AB, heterokaryon AB.

To test if regenerants of protoplasts released from multiple generations of SPRs may contain B type homokaryon, from 3rd to 6th generation, 5 SPRs each were used to produce SPRs and their karyotic nature was determined. All the SPRs contained A type homokaryon and heterokaryon AB with the exception of no. 5 of the 6th generation of SPR which gave rise to only heterokaryon AB (Table 6). To test if B type nuclei may require larger hole for exit from mycelia, protoplasts were obtained from mycelia of parental isolate and different generations of SPRs after repeated enzyme treatments. Even after the mycelia were treated with lytic enzymes 4 times, the protoplasts released were either A type homokaryon or heterokaryon AB (Table 7).

**Table 6:** **Karyotic nature of regenerants of protoplasts of** ***Rhizoctonia solani*** **AG1-IC isolate CH-1 released from six generations of protoplast regenerants**

Origin of protoplasts	Karyotic nature*(no.)			Total
	A	B	AB	
Parent	12	0	4	16
2nd generation	4	0	8	12
3rd generation				
1	3	0	7	10
2	2	0	8	10
3	2	0	8	10
4	2	0	8	10
5	6	0	4	10
4th generation				
1	4	0	6	10
2	2	0	8	10
3	2	0	8	10
4	4	0	6	10
5	6	0	4	10
5th generation				
1	4	0	8	12
2	4	0	8	12
3	2	0	10	12
4	3	0	9	12
5	5	0	7	12
6th generation				
1	4	0	8	12
2	1	0	11	12
3	1	0	11	12
4	1	0	11	12
5	0	0	12	12
Total	74	0	174	248

*A, homokaryon A; B, homokaryon B; AB, heterokaryon AB.

**Table 7:** **Karyotic nature of regenerants of protoplasts of** ***Rhizoctonia solani*** **AG1-IC isolate CH-1 released from mycelial mat of six generations of protoplasts treated with enzymes 2 to 4 times**

Origin of protoplasts and no. of enzyme treatment	Karyotic nature*(no.)			Total
	A	B	AB	
Parent				
2	1	0	18	19
3	0	0	12	12
2nd generation				
3	0	0	4	4
4	8	0	8	16
3rd generation				
2	0	0	20	20
4	0	0	20	20
4th generation				
3	6	0	14	20
4	3	0	17	20
5th generation				
3	9	0	11	20
4	13	0	7	20
6th generation				
2	7	0	13	20
4	3	0	17	20
Total	50	0	161	211

*A, homokaryon A; B, homokaryon B; AB, heterokaryon AB.

### New variant formation

Ranging from 10 to 30 single hyphal tip isolates were obtained from tufts resulting from three independent pairings between homokaryon A and heterokaryotic parent. Colony morphology of these isolates was mostly of the heterkaryotic parent type. However, colony morphology of about 7 to 40% of single hyphal tip isolates was different from homokaryon A and heterokaryotic parent, and appeared to be a new variant (Table 8). The new variants did not form tuft when paired with either homokaryon A or heterokaryotic parent. When protoplasts were obtained from the variants homokaryon A, heterokaryotic parent or the variant type of colony morphology was found among the protoplast regenerants of each variant (Table 9). Homokaryon B was not detected as before.

**Table 8:** **Colony morphology of single hyphal tip isolates from tufts resulting from parings between homokaryon A and heterokaryotic parent of** ***Rhizoctonia solani*** **AG1-IC isolate CH-1**

Parings	Colony morphology*(no.)			
	A	B	AB	Variant
1	0	0	12	8
2	0	0	28	2
3	0	0	8	2

*A, colony morphology of A type homokaryon; B, colony morphology of B type homokaryon (not found); AB, colony morphology of heterokaryon; variant, colony morphology different from A type homokaryon and heterokaryon AB type.

**Table 9:** **Colony morphology of protoplast regenerants of variants from tufts resulting from parings between homokaryon A and heterokaryotic parent of** ***Rhizoctonia solani*** **AG1-IC isolate CH-1**

Variants	Colony morphology*of protoplast regenerants (no.)			
	A	B	AB	Variant
1	10	0	2	8
2	6	0	9	5
3	18	0	0	2
4	14	0	2	4

*A, colony morphology of A type homokaryon; B, colony morphology of B type homokaryon (not found); AB, colony morphology of heterokaryon; variant, colony morphology different from A type homokaryon and heterokaryon AB type.

**Figure 1:**
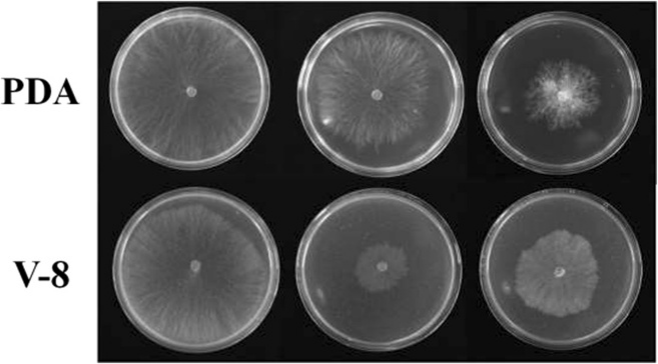
**Colony morphology of heterokaryotic parent (left), homokaryon A of protoplast regenerant (middle), and variant (right) resulting from pairing between heterokaryotic parent and homokaryon A of** ***Rhizoctonia solani*** **AG1-IC isolate CH-1 incubated on PDA (top row) or V-8 agar (bottom row) at 24°C for 36 h.**

On PDA, the heterokaryotic parent grew faster than the homokaryon A and the variant. The variant was the slowest in growth, about two times slower than the parent. On V-8 agar, the heterokaryotic parent also grew faster than the other two. However, the homokaryon A became the slowest in growth, only about one third of the parent (Table 10, Figure 1).

**Table 10:** **Average of linear growth of heterokaryotic parent, homokaryon A and variants of** ***Rhizoctonia solani*** **AG1-IC isolate CH-1 on PDA or V-8 agar**

Isolates	Linear growth*(mm/36 h)	
	PDA	V-8 agar
Heterokaryotic parent	51 ± 2 a	43 ± 1 a
Homokaryon A	35 ± 3 b	13 ± 2 c
Variant	19 ± 3 c	21 ± 3 b

*Data ± standard deviations represents the means of nine replicates. Values followed by the same letter in the same column are not significantly different using Tukey’s significant difference test at P = 0.05.

## Discussion

Confirmation of homokaryons of *R. solani* obtained through studies of basidiospores has been reported (Whitney and Parmeter [1963]; Carza-Chapa and Anderson [1966]; Anderson et al. [1972]; Adams and Butler [1982]) because not all basidiospores of this fungus are mononucleate (Flentje et al. [1963]; Anderson et al. [1972]). Since not all protoplasts of *R. solani* are mononucleate (Hashiba and Yamada [1982]; Phillips [1993]; Liu et al. [2010]), confirmation of karyotic nature of SPRs was also performed in this study. The result showed that the tuft formation method for determining the karyotic nature of SPRs is reliable.

Inability to obtain B type homokaryons from regenerants of protoplasts released from mycelia with various kinds of treatments suggests that the B type nuclei are probably defective and are dependent on A type nuclei for their multiplication. This may explain why SPRs contained only A type homokaryon and heterokaryon AB. Protoplasts containing only B type nuclei are probably not viable. The observation that only 48-79% protoplasts of this fungus were able to regenerate (Liu et al. [2010]) is compatible with this explanation. To our best knowledge, this is the first report of a heterokaryotic *R. solani* strain carrying a defective type of nuclei. The molecular difference between the defective and the normal nuclei and the effect of the defective nuclei on the pathogenicity of this fungus remain to be investigated.

When the heterokaryotic parent was paired with the homokaryon A, a variant was isolated from the tuft. The variant isolates did not form tuft when paired with the heterokaryotic parent or homokaryon A. The mechanism leading to such phenomenon remains to be investigated. When protoplasts were obtained from the variant isolates, the regenerants gave rise to heterokaryotic parent, homokaryon A or the variant, indicating that they belong to a new kind of heterokaryon.

## Conclusion

Inability of isolate CH-1 of *R. solani* AG1-IC to produce homokaryon B despite numerous attempts may suggests that B type nuclei are probably defective and are dependent on A type nuclei for their multiplication. This is the first report of a heterokaryotic *R. solani* strain carrying a defective type of nuclei. From this study a new variant which is a new kind of heterokaryon was also obtained from the tuft resulting from the pairing between the homokaryon A and the parental heterokaryon AB.

## Authors’ contributions

WHK conceived and designed the experiments, and wrote the manuscript. YNT performed the experiments. Both authors read and approved the final manuscript.

## Authors’ original submitted files for images

Below are the links to the authors’ original submitted files for images. Authors’ original file for figure 1
