# Supplementary figures and images for: A new variant produced by Rhizoctonia solani AG1-IC isolate CH-1 with a new type of nuclei
Source: Bot Stud. 2014 Sep 25;55:69. doi: 10.1186/s40529-014-0069-z (PMC5432740; doi:10.1186/s40529-014-0069-z)

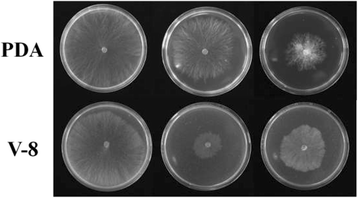

Supplement: Supplementary file 1 — Authors’ original file for figure 1 [file 40529_2014_9069_MOESM1_ESM.gif]
